# Cerebral haemodynamics during simulated driving: Changes in workload are detectable with functional near infrared spectroscopy

**DOI:** 10.1371/journal.pone.0248533

**Published:** 2021-03-12

**Authors:** Peter M. Bloomfield, Hayden Green, Nicholas Gant

**Affiliations:** 1 Department of Exercise Sciences, The University of Auckland, Auckland, New Zealand; 2 Centre for Brain Research, The University of Auckland, Auckland, New Zealand; Tokai University, JAPAN

## Abstract

Motor vehicle operation is a complicated task and substantial cognitive resources are required for safe driving. Experimental paradigms examining cognitive workload using driving simulators often introduce secondary tasks, such as mathematical exercises, or utilise simulated in-vehicle information systems. The effects of manipulating the demands of the primary driving task have not been examined in detail using advanced neuroimaging techniques. This study used a manipulation of the simulated driving environment to test the impact of increased driving complexity on brain activity. Fifteen participants drove in two scenarios reflecting common driving environments differing in the amount of vehicular traffic, frequency of intersections, number of buildings, and speed limit restrictions. Functional near infrared spectroscopy was used to quantify changes in cortical activity; fifty-five optodes were placed over the prefrontal and occipital cortices, commonly assessed areas during driving. Compared to baseline, both scenarios increased oxyhaemoglobin in the bilateral prefrontal cortex and cerebral blood volume in the right prefrontal cortex (all *p* ≤ 0.05). Deoxyhaemoglobin decreased at the bilateral aspects of the prefrontal cortex but overall tended to increase in the medial aspect during both scenarios (both *p* ≤ 0.05). Cerebral oxygen exchange significantly declined at the lateral aspects of the prefrontal cortex, with a small but significant increase seen in the medial aspect (both *p* < 0.05). There were no significant differences for oxyhaemoglobin, deoxyhaemoglobin, or cerebral blood volume (all *p* > 0.05). This study demonstrates that functional near infrared spectroscopy is capable of detecting changes in cortical activity elicited by simulated driving tasks but may be less sensitive to variations in driving workload aggregated over a longer duration.

## Introduction

Driving is a complex and, at times, highly complicated task performed daily by many adults. For the safe operation of motor vehicles, a driver must be able to perform decision-making, plan routes, and react to road conditions or hazards, all whilst physically controlling the vehicle in a safe manner [1]. Therefore, it is critical that drivers have sufficient cognitive resources to perform these tasks correctly, safely and, if required, rapidly. If insufficient cognitive resources are available for driving, it is likely that a decline in driving performance will occur, increasing the risk of accidents [2–4]. It is possible to assess the cognitive demands whilst driving using both psychological and physiological assessments and examining its influence on driving behaviour [5–7]. Additionally, many studies have investigated the effects of task load on driving performance [2, 3, 6, 8, 9]. These protocols typically alter cognitive workload using an unnaturalistic manipulation of the driving environment. Currently, the effects of different driving environments *per se* on cognitive workload have been not been well quantified.

Cognitive workload during driving is most commonly assessed/manipulated with a secondary task that is performed whilst operating the vehicle. Declines in secondary task performance are attributed to increases in cognitive workload, whereas changes in driving performance with the manipulation of the secondary task’s difficulty reflect the effect of cognitive load on driving performance. For example, numerous studies have used secondary tasks such as n-back [6, 8, 10] and surrogate in-vehicle information systems (SIVIS) tasks to manipulate cognitive workload [3, 9], demonstrating practical outcomes, such as an increase in the difficulty of the secondary task leading to an increase in dangerous driving behaviour. Although this can provide valuable information on the effects of available cognitive resources for driving, the ecological validity of secondary tasks that must be performed in conjunction with driving is not optimal, as such activities are unlikely to occur in most valid driving scenarios. In contrast, driving itself involves many neurocognitive domains and, therefore, altering the primary driving task alone would be expected to provoke differences in cognitive requirements, measurable as altered brain activation, independent of a secondary task [11, 12]. Foy et al. [7] were the first to investigate the effect of manipulating the demands of the driving *per se* on brain activity. The study used four scenarios with varying densities of traffic; participants were required to overtake differing numbers of cars in each scenario. Activity in the prefrontal cortex increased during overtaking, indicating a greater cognitive demand. This activation pattern was age-dependent, with younger drivers showing decreased activation compared to older drivers. Foy et al. manipulated a single component of the driving task and the effect of a more complete manipulation of the driving task on brain activity is yet to be determined.

Functional near infrared spectroscopy (fNIRS) is a non-invasive and non-distracting neuroimaging technique that measures haemodynamic changes to quantify cortical activity [13, 14]. fNIRS can measure brain activity in similar applications to functional magnetic resonance imaging (fMRI); more specifically, changes in deoxygenated haemoglobin have a close correlation to the blood-oxygen-level-dependent (BOLD) signal seen in fMRI [15, 16], whilst changes in oxygenated haemoglobin also have a correlation with the BOLD signal. Numerous studies have used fNIRS to assess changes in cortical activation using secondary cognitive tasks [6, 14, 17]; however, the effects of manipulating the driving environment alone have not been investigated in detail. Using fNIRS, this study aims to extend the work of Foy et al. [7] and determine how a more complete manipulation of the primary task affects cortical activity. This aim will be achieved by using fNIRS to quantify and contrast cortical activity during simulated driving in two scenarios of differing complexities. We hypothesised that the sensitivity of the fNIRS array would be capable of detecting differences in cognitive activity between the two scenarios. These differences would be seen as changes in the cerebral haemodynamics and cortical activation patterns in key brain areas.

## Methods

### Participants and study design

Fifteen healthy adults (4 male, 11 female) with a mean age of 22 years (range 21–24 years) volunteered to participate. Participants were in good health, and in possession of a full New Zealand driver’s licence; holding this licence required the participant to have at least 18 months driving experience. Participants attended the laboratory for one session. Ethical approval for this study was granted by the University of Auckland Human Participant Ethics Committee (ethics approval number: 021060) in accordance with the Declaration of Helsinki; all participants gave written informed consent before participating in the study.

### Driving simulator

A low-to-moderate fidelity fixed-base STISIM 300WS simulator (Systems Technology Incorporated, USA) was used. The apparatus simulated an automatic transmission vehicle, incorporating an accelerator pedal, brake pedal, steering wheel and turn signal lever. The simulation was displayed across three LCD monitors (Dell, 21”, total display resolution: 3840 x 1020 pixels) positioned 90 cm away from the participants. All simulator controls were positioned to suit the participant.

### Driving scenarios

**Fig 1:**
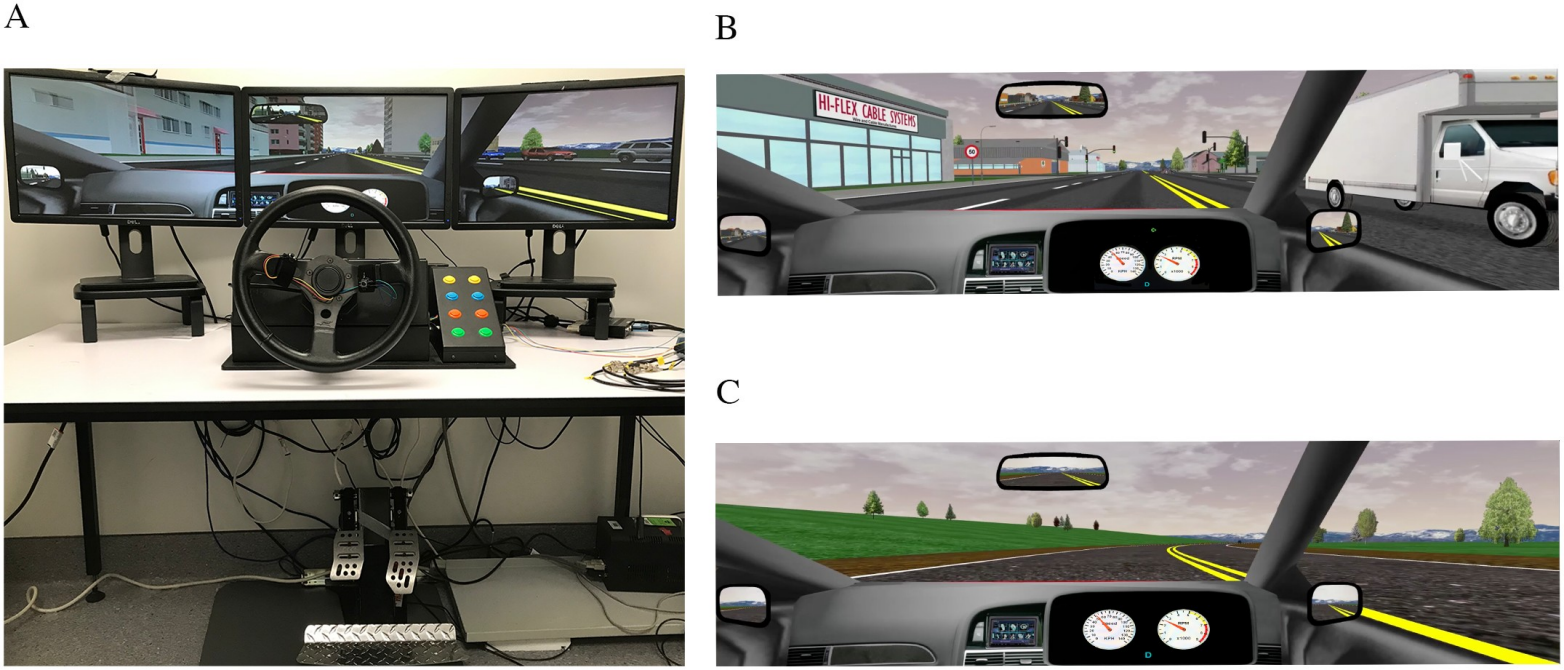
Driving simulator equipment configuration. A. An overview of the simulator composed of three-monitor display positioned approximately 90 cm from the participant, steering system, and pedals. B. An example of the high cognitive load scenario in an urban environment with a high density of buildings and traffic. C. An example of the low cognitive load scenario in a rural environment with a low density of buildings and traffic.

Participants completed rural and urban scenarios, hereafter termed “low” and “high” cognitive workload scenarios, respectively. Scenarios were completed in a randomised order. Participants were instructed to follow the New Zealand road rules at all times during both scenarios. The low load task involved driving on typical New Zealand rural roads. The route length was 20.1 km, the maximum speed limit was 100 km.h^-1^ and there were no intersections, sharp corners, buildings, or pedestrians. There was little traffic present (2 cars.min^-1^). In contrast, the high load task involved driving in a moderately built-up cityscape (21 buildings.min^-1^, ranging from residential houses to larger commercial buildings) containing heavier traffic (~16 cars.minute^-1^), pedestrians (17 pedestrians.min^-1^), intersections (12 total), and roadworks (3 total) consisting of a lane closed for approximately 600m using traffic cones surrounding trucks parked in the closed lane. The journey was 9.2 km long with a maximum speed limit of 50 km.h^-1^. Both scenarios were intended to take approximately 720 seconds to complete. Participants were directed through 12 intersections (4 left, 4 right and 4 straight ahead) via audio navigation cues built-in to the scenario. Failing to follow a navigation command caused the road to be reset in a manner unidentifiable to the participant, meaning the remainder of the route was unaffected by navigational errors. As there was no following traffic, participants were not required or instructed to check their blind spots. The driving simulator and examples of the two scenarios are shown in Fig 1.

### Functional near infrared spectroscopy

**Fig 2:**
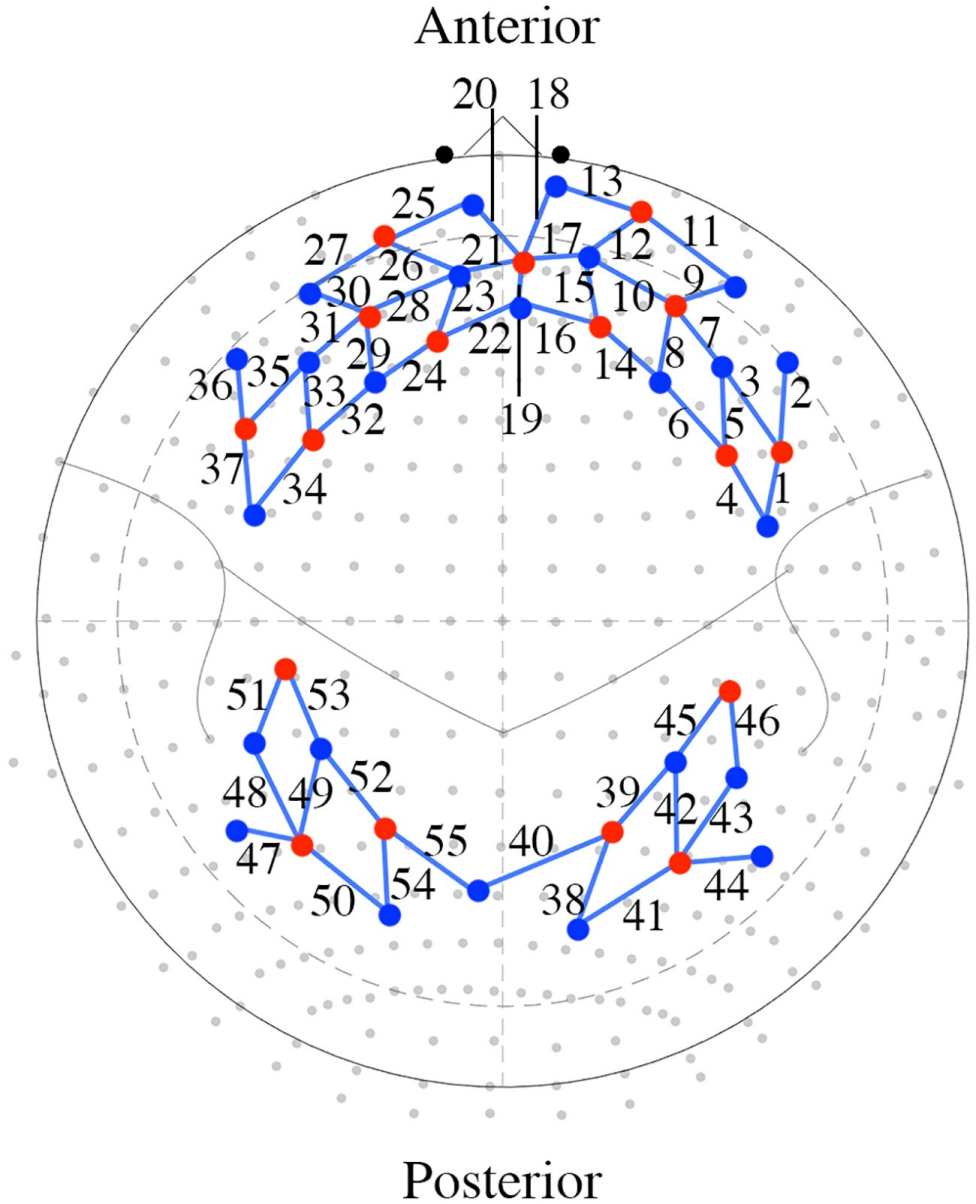
fNIRS optode layout. An anterior and posterior view of the optode montage mapped onto a template brain. Red dots denote a channel source and blue dots represent a channel detector. The blue line connecting a source and detector represents a measured channel; each channel is numbered. Note that the labels for channels 18, 19, and 20 are displaced; a straight black line indicates the position of each channel.

Cerebral haemodynamics were monitored using multi-channel functional near infrared spectroscopy or fNIRS (Brainsight, Rogue Research, Quebec, Canada). Sources and detectors were placed to measure cortical areas of the left and right prefrontal cortex, frontal cortex, parietal lobe, and occipital lobe (Fig 2). Chosen for their role in planning, execution, and interpreting visual input, these are commonly assessed areas in driving simulator paradigms [6, 7, 14, 18].

Fifty-five channels, comprised of 17 sources and 24 detectors, were used to measure both hemispheres of the frontal and occipital cortex, with an interoptode distance for all measured channels between 2.8 and 3.5 cm [19]. A neuronavigation system (Brainsight, Rogue Research, Quebec, Canada) and 3D position sensor (Polaris Vicra, NDI Medical) were used to register the optode layout onto the head of each participant and then project the optode array onto an MNI-152 brain atlas for analysis [20, 21]. Infrared wavelengths of 705 nm and 830 nm were used at 20 mW of power. fNIRS data were sampled at 10 Hz in the Brainsight software (Brainsight, Rogue Research, Quebec, Canada.). A resting baseline measure, lasting approximately 2 minutes, was taken before participants started any driving task. Participants were seated for approximately 10 minutes before the baseline measure was recorded.

#### Analysis of fNIRS data

Each channel was visually inspected for signal quality; channels with non-pulsatile signals or with excessive motion artefacts were removed from analyses, additionally, channels with a coefficient of variation (standard deviation divided by the mean) greater than 30% were also discarded. This coefficient of variation value is higher than other values reported in the literature [22, 23] but was intended to prevent the unnecessary removal of channels. Intensity-time data were converted to changes in optical density [24]. A bandpass filter (*f*_c_ = 0.01 Hz to 0.09 Hz, hmrBandpassFilt, Homer2) and principal component analysis filter (nSV = 1, hmrPCAFilter, Homer2) were applied to account for high frequency noise, baseline drift and the removal of motion artefacts [24, 25]. Based on a contemporary review [24] we selected these bandpass frequency values to remove physiological noise from heart rate (~ 1–1.3 Hz), the Mayer wave (~ 0.1 Hz), breathing rate (0.1–0.3 Hz), and very low frequency drift in the signal. The modified Beer-Lambert law was then used to convert changes in optical density to changes in concentration of oxyhaemoglobin and deoxyhaemoglobin [26, 27]. The differential pathlength factor (DPF) was kept constant at 5.93 as all participants were of a similar age [19]. All fNIRS signal analyses were completed in NIRS Toolbox [28]; however, the bandpass and PCA filter were applied from the HOMER2 toolbox using the “Run_HOMER2” function within NIRS Toolbox [29].

Haemodynamic responses were averaged over the full duration of each scenario. Cerebral blood volume (CBV), cerebral oxygen exchange (COE), oxyhaemoglobin (HbO) and deoxyhaemoglobin (HbR) were examined. COE and CBV were calculated using the following equations [25]: $\text{COE}=\frac{\Delta \text{HbR}-\Delta \text{HbO}}{\sqrt{2}}$ and $\text{CBV}=\frac{\Delta \text{HbR}-\Delta \text{HbO}}{\sqrt{2}}$.

Hypothesis tests to determine differences in cortical activation between the baseline, low-load, and high-load scenarios were completed with a linear mixed effects model, using Satterthwaite’s degrees of freedom method. All statistical analyses were completed in R (R 3.6.3) using RStudio [30]. The function “lmer” in the package lmerTest [31] was used to fit the model for HbO as follows: (HbO ~ condition + 1|subject), where “condition” was the scenario of interested (3 levels: baseline, low-load, and high-load), and “subject” was the intercept for each participant. This model was fitted to each channel, for each of HbO, HbR, CBV, and COE. If a statistically significant difference (*p* ≤ 0.05) between conditions was detected, pairwise comparisons using Tukey’s honestly significant difference (Tukey’s HSD) were applied to determine where the between-condition differences lay. *Post hoc* tests were applied using the function “emmeans” from the emmeans package [32]. Tukey’s HSD controlled for multiple comparisons within each channel; however, no method was used to control for the between-channel tests, as independence of channels could not be assumed. Data were anatomically mapped using xjView [33] and presented visually using Surf Ice [34].

## Results

The mean time taken to complete the high load scenario was 730 seconds (standard deviation = 23 seconds; range = 689 to 770 seconds) and the mean time taken to complete the low load scenario was 742 seconds (standard deviation = 15 seconds; range = 707 to 769 seconds). A paired sample t-test showed that this difference was not statistically significant (*p* = 0.13). Please refer to S1 File for these data.

A mean of 34 channels per participant covering the frontal lobe were deemed suitable for statistical analysis (range = 27 to 37 channels), with a mean of 14 participants included in each channel’s analysis (range = 12 to 15 participants). For a more detailed breakdown, please refer to S1 File. As the data recorded from optodes over the visual cortex contained too few valid channels (due to poor signal quality arising from the optode-skin connection) for meaningful analysis only the frontal lobe will be discussed here. For mean raw data please refer to S1 File.

**Fig 3:**
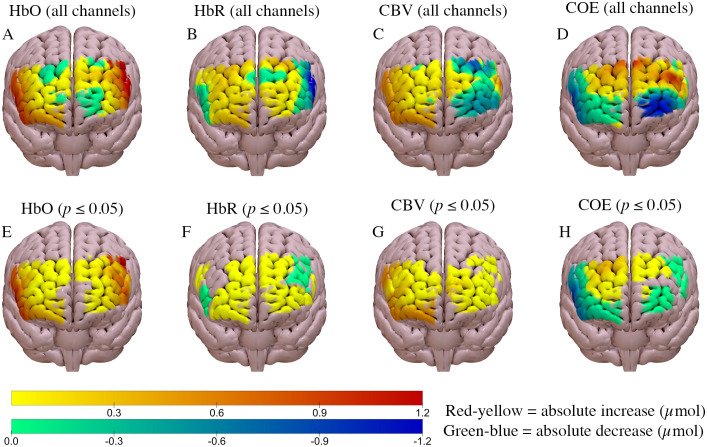
Haemodynamic response of the high load urban scenario compared to baseline. A, B, C, D = ΔHbO, ΔHbR, ΔCBV and ΔCOE, respectively for all channels. E, F, G, H show data only for channels with statistically significant differences from baseline (*p* ≤ 0.05) for ΔHbO, ΔHbR, ΔCBV and ΔCOE respectively. Anterior brain view is shown.

**Fig 4:**
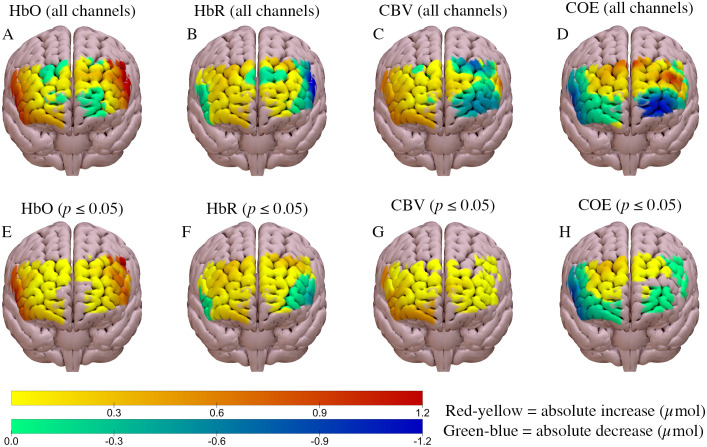
Haemodynamic response of the low load rural scenario compared to baseline. A, B, C, D = ΔCBV, ΔCOE, ΔHbO and ΔHbR, respectively. E, F, G, H show data only for channels with statistically significant differences from baseline (*p* ≤ 0.05) for ΔCBV, ΔCOE, ΔHbO and ΔHbR respectively. Anterior brain view is shown.

Regional brain activation increased in both the high and low load scenarios, compared to baseline (Figs 3E–3H and 4E–4H). Cerebral blood volume increased compared to baseline (*p* ≤ 0.05) in both scenarios, although the magnitude of change was slightly greater in the high load scenario (Figs 3G and 4G). Similarly, oxyhaemoglobin increased by a similar amount in both scenarios compared to baseline (*p* ≤ 0.05; Figs 3E and 4E). A statistically significant bilateral decline in cerebral oxygen exchange (*p* ≤ 0.05) was observed in both scenarios, compared to baseline (Figs 3H and 4H); in both scenarios this decline was greater in the right hemisphere.

**Fig 5:**
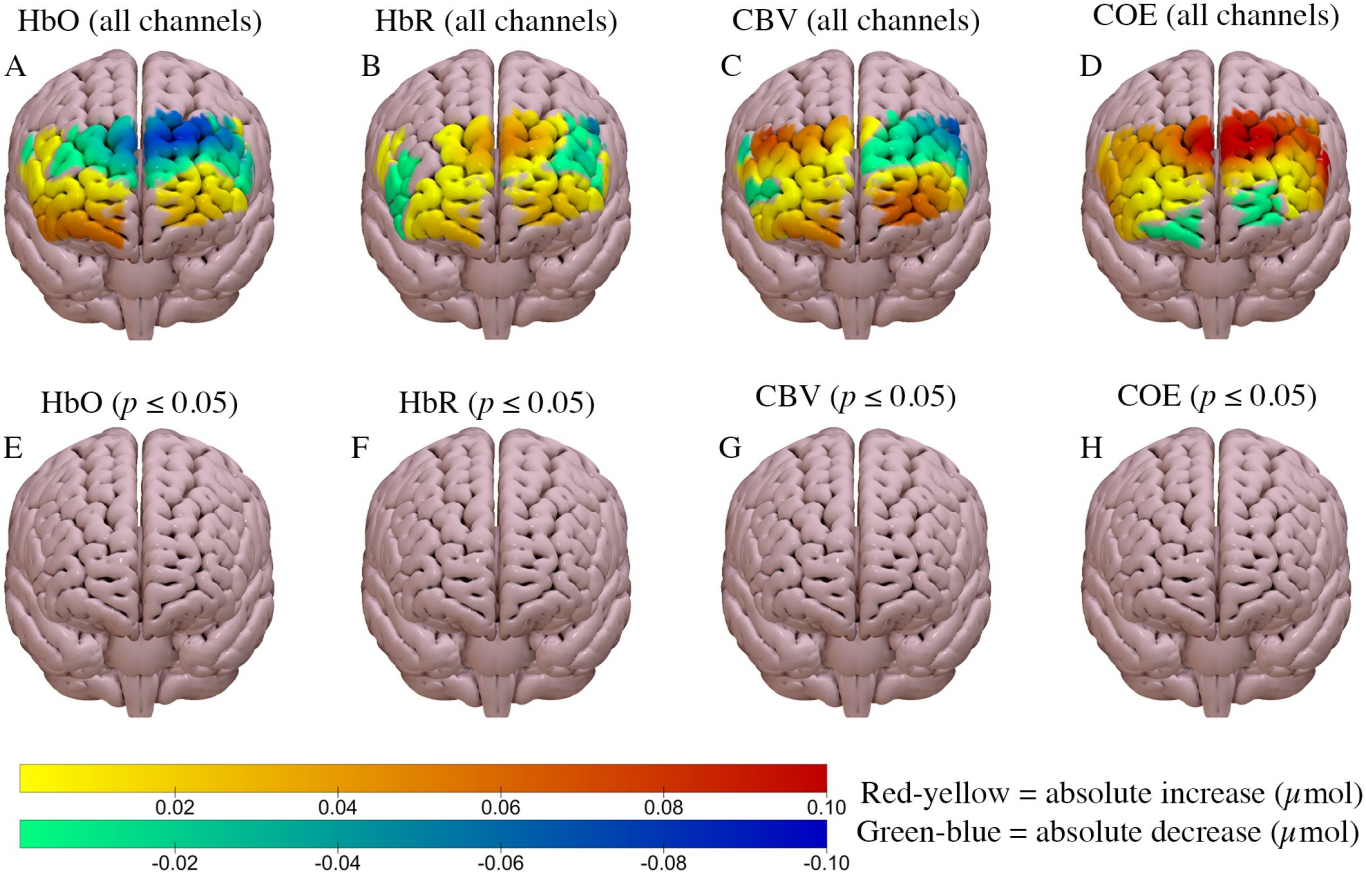
Between-scenario differences in the haemodynamic response. A, B, C, D = ΔHbO, ΔHbR, ΔCBV and ΔCOE, respectively. E, F, G, H show data only for channels with statistically significant differences from baseline (*p* ≤ 0.05) for ΔHbO, ΔHbR, ΔCBV and ΔCOE respectively. No statistically significant differences are present for panels E-H. Anterior brain view is shown. Note: The scale differs from that used in Figs 2 and 3.

Between-scenario differences were calculated by subtracting the response in the low workload task from the response in the high workload task. There were no significant differences for any of HbO, HbR, CBV, or COE (all channels *p* > 0.05; Fig 5E–5H, respectively).

## Discussion

This study demonstrates that fNIRS is sufficiently sensitive to detect activation of the prefrontal cortex whilst driving in both the high and low cognitive workload environments. Patterns of activation are similar to those seen in previous work by other groups [7, 35]. Oxyhaemoglobin significantly increased in the left and right lateral aspects of the prefrontal cortex during both high and low cognitive workload scenarios. Cerebral blood volume significantly increased in the prefrontal cortex in both scenarios, but to a slightly greater degree in the right hemisphere. Deoxyhaemoglobin showed a slight bilateral decline at the lateral aspects of the prefrontal cortex during both scenarios. In contrast, a modest increase in deoxyhaemoglobin was observed in the medial aspects during both scenarios, although the area was slightly larger in the low cognitive workload task. There was a significant decline in cerebral oxygen exchange in both left and right lateral aspects of the prefrontal cortex, but to a slightly greater degree in the right aspect; these changes were present in both low and high workload scenarios. When comparing the two cognitive loads, the activation patterns were very similar between scenarios. No significant differences were detected in changes of oxyhaemoglobin, deoxyhaemoglobin, cerebral oxygen exchange, or cerebral blood volume.

The activation in the right prefrontal cortex, shown by an increase in HbO and CBV, was similar to findings reported by previous studies [7, 36]. This was unsurprising given the role of the prefrontal cortex in controlling aspects of executive function critical for driving, including inhibition of inappropriate responses, appropriate response selection, attention, and control of cognitive workload [7, 36–39]. It is known that younger drivers frequently show decreased activation of the prefrontal cortex compared to older drivers [7] as this brain area is one of the last to mature; therefore, our study may have been limited by the relatively young age of participants. A sample with a greater age range may have shown a greater level of prefrontal cortex activation throughout both scenarios.

The significant decrease in deoxyhaemoglobin at the lateral aspects of the prefrontal cortex during both the high and low workload task (Figs 3D and 4D) was expected as neurovascular coupling is thought to result in decreased deoxyhaemoglobin at active brain sites [11]. The respective changes in oxyhaemoglobin and deoxyhaemoglobin likely contributed to the significant decline in cerebral oxygen exchange at the lateral aspects of the prefrontal cortex in both scenarios (Figs 2B and 3B). A decreased COE value is indicative of increased oxyhaemoglobin, decreased deoxyhaemoglobin, or both [25]. These changes are suggestive of increased brain activation in the lateral aspects of the prefrontal cortex, which is expected with the increase in cognitive demand from driving. However, it was hypothesised that greater changes would occur at the medial and frontal aspects of the prefrontal cortex, similar to previous studies, as these brain areas are thought to be more involved in driving.

The similarity in activation patterns between scenarios suggests that both tasks placed a comparable demand on cognitive resources. While this was unexpected, there are some possible explanations. As changes in cerebral haemodynamics were averaged over the entire scenario, the haemodynamic response to intersections, obstacles and the audio navigation system may have been reduced as these components only accounted for a small fraction of the total task. This may have rendered the two scenarios too similar for significant overall differences to be detected. Foy et al. [7] examined brain activity within an epoch around overtaking manoeuvres, which likely increased the specificity of their data. Our approach may be improved by using a paradigm that can examine the neurovascular response around points of interest, such as the approach to intersections or the identification of known hazards. An event related analysis has been previously conducted by Yoshino et al. [25] in highway driving; however, this was limited to acceleration, constant speed driving, deceleration, and a U-Turn. Whilst their results indicate that during a U-Turn, a relatively challenging aspect of driving, there is an increase in prefrontal cortex activation, this may not be reflective of driving challenges that can occur from events prior to a driver initiating the manoeuvre.

There are several limitations to this study. All participants were under the of 25 and the cohort included both male and female participants, Young males may exhibit poorer driving [40] and there was likely to be considerable variation in the level of maturation in the frontal lobe, increasing inter-subject variability.

While we attempted to account for, and remove, physiological noise via the bandpass filter we did not record simultaneous physiological measures, so it is not possible to confirm the presence or absence of some systemic physiological noise. Future studies could also use an accelerometer to measure head movement, thereby improving detection of motion artefacts.

## Conclusions

This study demonstrates that fNIRS is capable of detecting changes in cerebral haemodynamics caused by simulated driving tasks. We found that both high and low cognitive workload scenarios significantly altered cerebral haemodynamics compared to baseline, with corresponding regional increases in cerebral blood volume and oxyhaemoglobin at the right lateral prefrontal cortex. Future studies might examine brain activity around events within a driving simulation to elicit more specific responses. Findings from this study will be used to inform future study designs and analyses.

## Supporting information

S1 File Driving environment supplementary data. (XLSX)
